# In search of self after stroke: a longitudinal qualitative study in the context of client-centred rehabilitation

**DOI:** 10.1080/17482631.2023.2282513

**Published:** 2023-11-27

**Authors:** Anette Erikson, Maria Ranner, Susanne Guidetti, Lena von Koch

**Affiliations:** a Division of Occupational Therapy, Department of Neurobiology, Care Sciences, and Society, Karolinska Institutet, Stockholm, Sweden; b Health Medicine and Rehabilitation, Department of Health, Education and Technology, Luleå University of Technology, Luleå, Sweden; c Division of Occupational Therapy, Department of Neurobiology, Care Sciences, and Society, Karolinska Institutet, and Karolinska University Hospital, Women’s Health and Allied Health Professionals Theme, Stockholm, Sweden; d Division of Family Medicine and Primary Health Care, Department of Neurobiology, Care Sciences, and Society, Karolinska Institutet and Karolinska University Hospital, Theme Heart & Vascular and Neuro, Stockholm, Sweden

**Keywords:** Activities in everyday life, constant comparative method, the lived body, occupational identity, occupational therapy, participation

## Abstract

**Purpose:**

The aim was to illuminate the experiences of stroke survivors returning to everyday life in the year following stroke, in the context of client-centred rehabilitation.

**Methods:**

Four men who participated in a client-centred rehabilitation program were followed during the first year after stroke. Semi-structured interviews were conducted, allbut the first in the participants’ home. The data were analysed using a grounded theory approach.

**Results:**

The results revealed a process with the overarching core category; The “new” self becomes reality through challenging everyday experiences, and five main categories driving the process: 1) Striving for structure in a “new” chaotic world, 2) Homecoming an ambiguous experience, 3) Reaching the “new” self through reflections of self-understanding, 4) Socialising in new circumstances, and 5) Realising a new reality.

**Conclusions:**

While in hospital, stroke survivors can have unrealistic expectations. When at home they can begin to realise their actual capacity . To find a “new” self after a stroke can involve time-consuming and taxing processes of reflections of self-understanding. Engagement in self-selected meaningful and valued activities can support stroke survivors’ reconstruction of the “new” self but not all stroke survivors may succeed in finding their “new” self during the first year after stroke.

## Introduction

Stroke, one of the leading causes of disability (Vos et al., 2017), has a sudden onset and is a stressful event, both for the stroke survivors and their family (Wissel et al., 2013). A stroke can lead to a wide range of symptoms such as motor- and cognitive impairment, fatigue, and depression that can have an impact on the individual’s ability to resume previous activities and roles (Arntzen et al., 2015). Some differences between men and women in the recovery after stroke have been reported where women have poorer functional recovery, lower quality of life and higher incidence of depression (Rexrode et al., 2022). Improvements in acute medical care in high-income countries have led to a reduction in mean length of hospital stay in the stroke unit (Tistad et al., 2012).

The recovery process after stroke is long and complex, as reported in qualitative and quantitative studies. Qualitative studies have shown that many stroke survivors experience a loss of self and identity. Studies one year after stroke have revealed that individuals need to establish self-confidence (Horne et al., 2014) and reconstruct the embodied self for positive quality of life experiences (Pedersen et al., 2019). A variation in the personal, social, and economic impact, emotional distress and fatigue, was reported in a national survey of community re-integration in the first 5 years after stroke (Walsh et al., 2015b). Appropriate support from family, friends, and community was of importance for the individual’s ability to overcome emotional challenges and for community re-integration during the first year (Walsh et al., 2015a, 2015b). To return to work has also been presented as a continuous struggle, with invisible impairment many years after stroke (Palstam et al., 2018). One study concluded that the central process of adjustment was the reconstruction of an occupational identity (Waldner & Molineux, 2017) i.e., a positive view of oneself as a person who is able to be engaged in meaningful and valued activities.

Longitudinal qualitative studies have shown that 5–15 years after stroke people still experience difficulties in their everyday life (Arntzen et al., 2015; Erikson et al., 2010, 2016; Norlander et al., 0000, 2018; Singam et al., 2015; Williams & Murray, 2013). More than 10 years after stroke, they still struggled to reclaim their former existence to find meaning in their lives (Erikson et al., 2016). Experiences of a totally changed life have been reported (Williams & Murray, 2013), as well as a long-lasting struggle to overcome the tension between the lived body, participation in everyday life, and a sense of self (Arntzen et al., 2015). Nevertheless, studies have also shown that participation can be attained after stroke and it is associated with several personal and environmental conditions (Norlander et al., 2018) and that a return to pre-stroke levels of participation can be achieved despite impairments (Singam et al., 2015).

Moreover, it has been shown that both stroke survivors and their partners were involved in the process of adapting and rebuilding a post-stroke life and identity (Lou et al., 2017).

Rehabilitation after stroke can be viewed as a process in which the stroke survivors negotiate with healthcare providers on realistic goals and activities for their rehabilitation (Bendz, 2000). While stroke survivors may view themselves as the individuals they were before stroke, in the medical records they can be described by health care professionals as someone of a certain age with certain impairments and activity limitations (Bendz, 2000). Hence, there may be a difference in point of departure and the challenge how goals can be set, and experienced as relevant by both the stroke survivor as well as by healthcare professionals has been recognized (Bendz, 2003).

Empirical evidence supports involvement of stroke survivors in the planning and decision of their own care and rehabilitation (Kristensen et al., 2016). Such involvement aligns with the concepts patient-centred, person-centred or client-centred care and rehabilitation, which is also advocated in policy documents (Swedish Agency for Health and Care Services Analysis, 2015; WHO, 2007). These concepts are sometimes used interchangeably. In this study, the concept client-centred is used implying rehabilitation tailored to the client’s ability and perceived needs, and which takes the client’s lived experiences as the point of departure (Guidetti et al., 2022). The concept client-centred emanates from Carl Rogers who emphasized that therapeutic relationships require the understanding that the clients are the experts and the therapists tools to support the clients in finding solutions (Rogers, 1946). It has been shown that person- or client-centred care is not well implemented in rehabilitation (Yun & Choi, 2019) and how stroke survivors experience their everyday life and functioning at different times after a stroke after having received client-centred rehabilitation is largely unknown.

Therefore, the aim of this study was to illuminate the experiences of stroke survivors returning to everyday life in the first year after stroke, in the context of client-centred rehabilitation.

## Materials and methods

### Participants and study context

Participants in this study included four individuals, all men who had had a stroke. They were participants in a study (Clinical Trials.gov identifier: NCTO 1,418,575) in which a “client-centred activities of daily living” (CADL) program was evaluated in a randomized controlled trial (RCT) (Bertilsson et al., 2014; Guidetti et al., 2015), along with nested qualitative studies of the experiences of occupational therapists (OTs) (Ranner et al., 2016), of stroke survivors (Ranner et al., 2019) and their partners (Bertilsson et al., 2015). Individuals able to express experiences of rehabilitation, and of everyday life while resuming daily activities during the first year after stroke, were eligible to participate. The OTs who delivered the CADL were asked to suggest clients who could participate. The participants received written and verbal information about the study, and informed consent was obtained just before the first point of data collection. The researchers involved did not have any previous connection to the participants. Detailed descriptions of the RCT study context have previously been presented (Bertilsson et al., 2014). Stroke survivors eligible to be included were: 1) treated for acute stroke in a stroke unit less than 3 months ago, 2) dependent in at least two activities of daily living (ADL), 3) able to understand and follow instructions, and 4) not diagnosed with dementia.

### The client-centred ADL program

The aim of the CADL was to enable agency in daily activities and participation in everyday life. Agency was defined as the experience of being responsible for one’s own actions and their outcomes with an element of choice and the power to act.

The content of the CADL provided a structure for how to discover and resolve problems based on the clients’ lived experiences in everyday activities. The CADL was to be performed in close collaboration between the client and the OT, and was to be adjusted to the individual’s ability, motivation, and needs. The CADL comprised nine components presented in Table I. The aim was to create a relationship with the client based on trust. Subsequently, the client and the OT together identified goals based on activities that the client wanted and needed to perform. To reach these goals, problem-solving strategies were identified. Then, strategies to enable successful performance of a chosen activity were identified and put into practice. Lastly, the clients and the OTs together reflected and evaluated goal attainment. Thereafter, all strategies used by the client were scrutinized to facilitate transfer of learning and skills to activities and situations outside therapy.

**Table I.** The nine components of the client-centred activities of daily living (CADL)intervention.

The 1^st^ component was the first meeting with the client which aimed to create a relationship between the client and the occupational therapist (OT) based on trust. In the 2^nd^ component the client was observed when performing an activity. A combined strategy of collecting information from conversations and observations in actual practical situations was used by the OT to create a picture and an understanding of the client’s situation. The 3^rd^ component entailed scoring the activity together with the client using the Canadian Occupational Performance Measure (COPM) and to identify problem-solving strategies for successful performance of the chosen activity. In the 4^th^ component the client, together with the OT formulated goals by using the COPM. In the 5^th^ component the OT was using a Goal-Plan-Do-Check strategy to guide the client in the discovery and formulation of an intervention plan to meet the goals. In the 6^th^ component the client was introduced to using a diary as a structure for training. In the 7^th^ component Reporting and involving others the OT supported the client to inform persons related to them, e.g., significant others, staff members at the rehabilitation clinic about their goals and planned strategies for the CADL intervention. In the 8^th^ component the client was training to perform and integrate activities by using the global problem-solving strategy as a structure for doing in a new way. The clients practiced the chosen activities named in the intervention plan using the planned strategies, both on their own and with the OT. The 9^th^ component included a session in which the OTs and the clients evaluated the goals, reviewed the strategies, and then formulated new goals to facilitate transfer of learning to activities and situations outside therapy.

### Data collection

Longitudinal data were collected by semi-structured interviews at three (*n* = 3) or four (*n* = 1) occasions during the first year after stroke. Interview guides adapted to the point in time for data collection were used. The questions were asked in a manner to generate rich data. At each interview, the participants were asked to describe their everyday life, and experiences of daily activities. Some general questions were used at each point of data collection e.g., please tell me what you do during an ordinary day. Whereas other questions were specific e.g., could you please tell me about your experiences of the rehabilitation you receive/have received. Follow-up questions were used to deepen the participants’ narratives when needed e.g., could you please tell me more about how you felt then. The first interview was conducted in the hospital and the following in the participants’ homes. Interviews were originally planned to be conducted four times, during and after the CADL program was finished, and at 6 and 12 months. The length of the participants’ hospital stays varied; consequently, the time between the first and the second interviews also varied. For three participants (A, B, and D) the post-CADL interview coincided in time with the six-month follow-up. It was considered too close in time for two interviews, as the participants would not have additional experiences to reveal. Hence, four interviews were conducted with participant C according to the original plan; for participants A, B and D, the third interview was conducted at 12 months. Consequently, quotations in findings were labelled as follows: 1=during CADL, 2=post-CADL, 3=at 6 months, and 12 months. All 13 interviews were conducted by the second author. Each interview lasted between 40 and 60 min and was digitally audio-recorded and transcribed verbatim. All interviews were conducted in Swedish, and quotations were translated into English after completion of the analysis.

### Data analysis

The first and last authors analysed the texts, following the flexible guidelines for grounded theory by Charmaz (Charmaz, 2006) which are based upon the constant comparative method (Glaser & Strauss, 1967). The data collection of the four participants was already completed as the analysis started. Hence, the cyclical process of collecting and coding data in grounded theory that usually begins after the first interview was not applied in this study. However, the structure of the data analysis followed the coding criteria according to grounded theory (Charmaz, 2006). In the initial coding, the transcribed interviews were analysed line-by-line. Similarities and differences in the data were identified and compared at each level of analysis using initial, focused, and axial coding. Questions raised during the initial coding were, for example, how the participants during the year experienced changes in the process. Examples could be what it meant to perform activities with others (rehabilitations staff, family, friends, and colleagues) in different places. Vivo codes during this stage of analysis helped to capture the participants’ meanings or experiences found during the initial coding. In the last stage of the analysis, axial coding refined the dimensions of the main categories by relating them to subcategories. The main category, conscious strategies, was, for example, finally related to the subcategories, concrete and mental strategies.

The first and last authors were engaged in the analysis and in refinements of the categories. To increase credibility, the second and third authors recurrently peer-examined and then all authors together discussed the analysis until agreement was reached.

The Regional Ethical Review Board in Stockholm approved the study (no. 2012/428–32).

## Results

**Figure 1.**
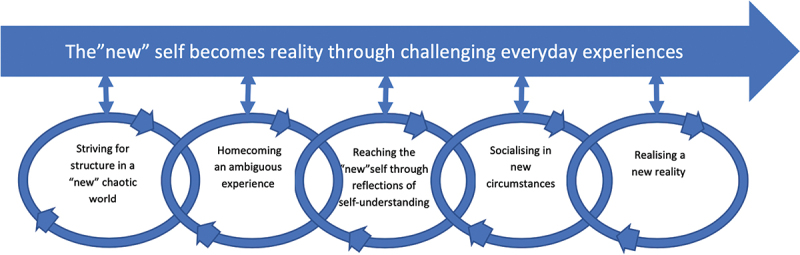
The “new” self becomes reality through challenging everyday experiences.

In Table II, an overview of the four participants’ characteristics is presented. They had all received their CADL-program as inpatients in the same hospital. The findings describe a process throughout the first year after stroke in the context of client-centred rehabilitation. Five categories with subcategories (in italics below) depicted in Figure 1, emerged from the analysis: 1. Striving for structure in a “new” chaotic world, 2. Homecoming—an ambiguous experience, 3. Reaching the “new” self through reflections of self-understanding, 4. Socialising in new circumstances, 5. Realising a new reality. From these categories, an overarching core category emerged; The “new” self becomes reality through challenging everyday experiences as depicted in Figure 1. There was a temporal process during the year as presented across the five categories. The temporal process was neither linear nor identical across the participants but captured their experiences during the year.

**Table II.** Overview of the participant characteristics.

ID #	Sex	Age	Living situation	Barthel Index before stroke^†^	Barthel Index after stroke^†^	Stroke hemisphere	Speech limitation after stroke
A	Male	61	Lived with wife	100	40	Left	None
B	Male	63	Lived alone	100	10	Left	Limited vocabulary
C	Male	60	Lived with wife	100	85	Left	None
D	Male	53	Lived with wife and children	100	30	Left	None

^†^Barthel Index, range 0–100

## Striving for structure in a “new” chaotic world

To initially land in a “new” world after stroke was a complex and taxing process. While in the hospital, relying on others was one way to strive for structure. Another way was to strive for structure by using conscious strategies, concrete in everyday activities and mental in preparation for coming home.

*Relying on others* (the rehabilitation staff) initially after stroke was a way to relate to oneself during rehabilitation. The participants seemed to see themselves as objects by completely entrusting themselves to the staff’s directives and decisions to regain their pre-stroke life, examples were as follows: “They have to curb me hard” (B1). “I will only do the exercises that are presented to me” (D1). The extent to which they submitted themselves to others can be exemplified by a participant who relied so much on others that he used “us” instead of “me” during training, which seemed to give him a sense of competence. “Now, we take on the underwear, now we wash,” (A1). He expressed that he did not feel involved in decision-making about his rehabilitation and demonstrated a frustrated sense of exclusion and uncertainty regarding the approaching hospital discharge. “But who the hell really decides what to do, who decides that I’m done?” (B1).

*Conscious strategies* both concrete and mental were applied by the participants while in the hospital. A way to get structure and to notice progress in the rehabilitation was to use concrete strategies in different activities. Examples of concrete strategies were to consciously calm down, or to write a diary to notice progress: “Just remember how you did in the beginning, when you were much worse, and you can see your progress” (C1). Teaching the left hand during ADL training was another concrete strategy. Indicating that the participant might have perceived his hand as an object rather than as a part of his body.

*Mental strategies*, while in hospital, were used to paint mental pictures when envisioning how activities could be transferred to various activities at home: “I think you could practice a lot at home; then we will see, try a little easier stuff” (A1). Playing an air guitar was another mental strategy used by a participant as a way of not having to be confronted with his true capacity: “I can practice, and I don’t need a guitar” (C1). Before coming home, the participants seemed to have difficulties to visualise what their actual capacity would be at home.

### Homecoming—an ambiguous experience

*The idealised image of coming home was shattered*, and there were feelings of disappointment and being left to oneself. The participants now had to adapt to their familiar environment under new circumstances. This was expressed as disappointments and distrusts in their abilities to practice at home, without receiving feedback from the rehabilitation staff: “It doesn’t work at home, I’ve told them, sometimes I can get started but it won’t be the same and I don’t see myself getting better or not” (A2). “I made progress, but now I have completely lost myself when I am on my own” (B2). It seemed that the idealised image of coming home was shattered and to some extent they felt lost in their new situation. Despite feeling lost, after some months the participants developed *an increasing awareness of the importance of contributing at home* expressed by one participant (C2) who contributed by assisting his wife by cleaning and cooking. As the year passed, his assisting at home developed into a habitual act. “I try to be up and running, clean a little, vacuum and dry, wash some clothes, cook some food because I am at home pretty much by myself” (C3). The activities in the home not only contributed to relieving others but also as for another participant to create structure: “Actually I am a bit of a night owl, but now I have better routines regarding when I go to bed and get up, the way people usually do” (D2). Thus, activities at home seemed to contribute to training, to organise everyday life, and to create structure.

### Reaching the “new” self through reflections of self-understanding

During the year, the participants made reflections of self, related to their new situation after stroke. Most of the participants (A, C, D) increasingly realised the impact of their stroke on their everyday lives. Self-understanding and time provided nuances, for example after some months at home a growing self-esteem was expressed. Some participants described themselves as unique regarding their rehabilitation process, which was described as “rapid” (C2) and by another participant as incredible: “It feels like you’re back, pretty much, with this self-esteem, and it does good to be able to do it yourself now. It is an incredible progress” (D2). In retrospect, another participant expressed a more nuanced perspective of the importance of the rehabilitation in the hospital. He did not identify himself as a typical person with stroke “no typical stroke case, it has gone so fast for me, so I am a bit surprised myself, it almost seems that they have never seen such a rapid rehabilitation” (C2). After some additional time, he (C3) described that it was necessary to reflect to see his own progress identifying himself as a person who had had a stroke. Time seemed to give three of the participants (A, C, D) perspectives through reflections of their progress after stroke. In contrast, the fourth participant (B) did not reach an understanding of his “new” self during the year and instead conveyed feelings of giving up.

### Socialising in new circumstances

Returning to their social life in private, and partly at work, in new circumstances during the year was a demanding process. Initially, there was a struggle with altered private relationships and a loss of spontaneity in life. There was an internal process in handling the complex relationship between work and private life. Towards the end of the year, returning to work was experienced as too exhausting to be realistic for all participants.

*A complex relationship in social life* was experienced during most of the year. Loss of spontaneity when socialising with others was an example of restrictions that required adaptation during the year, for example to choose simpler social activities than before stroke: “Out of the question for me to invite to a party but welcome to my home for some hot dogs instead” (C2). Another participant felt restricted because he could no longer drive to meet friends. Instead, he (D 12 months) eventually developed connections over the phone to his pre-stroke social network. The increased complexity in social life can also be exemplified by a participant who after 1 year said: “It is difficult to be married already, and on top of it all now you’re ill, too” (A 12 months). During the year, it appeared to be a constant struggle for the participants to socialise.

*An internal process between work and private life* developed during the year. Thoughts of work aroused different emotions. Initially after stroke, it was too complicated for the participants to consider their abilities in relation to their work situation. Throughout the year, stressful emotions were expressed when thinking of work, e.g., fear of going back since the work was experienced as too demanding, even bodily symptoms were described. A participant, who at the end of the year feared going back to work as work was too stressful for him, transferred the solution to his employer. He (D 12 months) was hoping his employer would solve his work situation. Another participant even described bodily symptoms when thinking of returning to work: “I have a little tension in my head: how will it be when I am not on sick leave anymore? I notice that I almost can’t get enough air, I feel so weak in my whole body” (C3). It appeared to be traumatic for him not to be able to explain his invisible disabilities at the workplace. After a year, daily activities at home had replaced his work, and he identified himself as retired: “So much vacuuming, then I cook and clean a bit everywhere, that’s my day, after all. I feel like a pensioner” (C 12 months). The participants´ thoughts of returning to work seemed to create stress throughout the year.

### Realising a new reality

One year after stroke, a realisation of a new reality could be found in most of the participants (A, C, D). They raised different existential reflections such as awareness of residual impairments, their own responsibility in rehabilitation, need for evidence to see progress, changed role in life and the importance of rehabilitation to understand the “new” life after stroke. One participant (A 12 months) reflected over the fact that he might never be fully recovered:” I may be clearer in the head now, in some ways. I wonder a bit myself what works” (A 12 months). He also made reflections about his own responsibility for his development and diverted his negative thoughts: “To get rid of a lot of these thoughts, you can’t be so negative all the time, what is the alternative really?” (A 12 months). He had realised that his initial goals were unlikely to be achieved and therefore did not set any new goals. Another participant realised that he needed tangible evidence to understand that he had made progress and did not want to be stigmatised in a sick role: “You need evidence to grasp the fact, that slowly but surely I will probably move forward. I do not like to talk about being ill. I want to be as usual” (C 12 months). One year after stroke another participant (D 12 month) reflected on the importance that the rehabilitation had had for his understanding of his “new” life after stroke:“It has greatly influenced one’s world of thoughts, providing insight, and thoughts that life is very fragile” (D 12 months).

In contrast to the participants above, one participant did not appear to experience any improvements or change in his perspective of his situation or to make progress in the recovery during the 1 year follow-up. Unlike the other participants, he did not seem to reconstruct a “new” self or an occupational identity. “I think I am worse, but they say I am not” (B 12 months). There seemed to be an imbalance between his perception of his ability and the view of others. For most of the participants (A, C, D) the process during the year after stroke seemed to have contributed to existential reflections about different aspects of life in realizing their “new” self after stroke.

## Discussion

To our knowledge, this is the first one-year longitudinal qualitative study in the context of client-centred rehabilitation of the experiences of stroke survivors who return to everyday life. The findings revealed one core category; The “new” self becomes reality through challenging everyday experiences. The discussion will for the most part follow the temporal process of the emerging categories as presented in the results.

While in the hospital, during the CADL the participants did not seem fully aware of their real capacity to perform activities. They instead appeared to rely on the healthcare professionals’ view of their capabilities (Kristensen et al., 2016). This is noteworthy considering that they all had received the CADL-program in which the participants and the OTs in partnership evaluated the participants’ activity performances and together set goals (Flink et al., 2016) and identified strategies for successful performance. Despite that they themselves while in hospital evaluated their own activity performance, they could not transfer what it would be like to perform the activities in their home environment. Their mental picture of their capacity did not seem to resonate with their actual performance. This gap between the participants’ perceived capacity and actual capacity may have contributed to disappointment and feeling lost. This finding is in concordance with similar findings in studies emphasizing the need to establish self-confidence (Horne et al., 2014) and reconstruction of the embodied self for positive quality of life experiences in the recovery process after stroke (Pedersen et al., 2019). Coming home included shifting experiences in which idealised expectations envisioned as part of mental strategies at hospital were not realized. High expectations of improvements upon return home have previously been described (Wottrich et al., 2012). It was not until the participants performed activities at home that they seemed to become fully aware and understood their actual competence. Doing activities to become familiar with the new body resonates with the philosopher Merleau-Ponty, who stated that the body is experienced from an internal perspective. Only when we perform activities do we gain experiences and become aware of bodily limitations (Meurleau-Ponty, 2002). A clinical implication is to encourage engagement in self-selected meaningful and valued activities to support stroke survivors’ reconstruction of the “new” self. Furthermore, there appears to be a need to improve the content of rehabilitation in the hospital after stroke to better assist the client in preparation of what it will be like to come home.

Socialising in new circumstances revealed an iterative process in which the participants negotiated the new circumstances for the complex relationship between work and private life. Socialising in private life could also include an increased awareness to contribute to the family and enable family members to continue their own activities. It seemed as if it was not until the participants started to find their self socially in private life that they increasingly reflected on their working life. In the beginning of the year, the participants seemed to push thoughts of work away as they did not seem able to relate the future to their “new” self. Thoughts of work appeared to be stressful, e.g., for not being able to explain invisible disabilities. Other research also shows that returning to work is a constant struggle and that invisible impairments can be a barrier for returning to work after stroke (Palstam et al., 2018). Recurrently thinking of returning to work seemed to make the participants gradually more aware of their bodily and mental limitations. These processes eventually appeared to lead them to recognising a new reality in which the participants in the present study seemed to consider themselves unable to return to work. Hence, in accordance with previous research, the participants initially related to their future based upon their former competence (Erikson et al., 2007) and gradually the home became a place with an increased meaning, both for socialising and being active (Erikson et al., 2010).

Towards the end of the year, three participants (A, C, D) could reflect on their “new” self and realise a new reality. The struggle to find a “new” self after stroke seemed to be demanding and challenging processes involving reflections of self-understanding. Over time nuances appeared as the participants gradually realised the stroke’s impact on their everyday lives and their identity as someone who had had a stroke. In the process of reaching this realisation, they conveyed existential reflections such as over the fragility of life, which has been reported to be of crucial importance after a stroke (Williams & Murray, 2013). Such reflections at one year could also be related to the initial rehabilitation as for participant D. One participant (B) didn’t seem to experience any improvements or positive changes in his situation, or to make progress in the recovery during the one-year follow-up. Unlike the others, he didn’t seem to reconstruct a new occupational identity. These findings are in agreement with longitudinal studies demonstrating the importance of establishing a positive self-belief (Erikson et al., 2016) and a need to reconstruct the embodied self for quality of life (Guidetti et al., 2007) and that these can be long-lasting processes (Arntzen et al., 2015; Erikson et al., 2016; Hole et al., 2014; Williams & Murray, 2013).

Despite that the categories depicted arduous and negative experiences, the participants also reported positive experiences that might be associated with the client-centred context in which the rehabilitation was initiated. One man, while inpatient, focused on his interest in music by playing air guitar. He continued throughout the year with his interest as it created meaning and supported his occupational identity. A clinical implication is to encourage engagement in self-selected meaningful and valued activities to support stroke survivors’ reconstruction of the “new” self.

The client-centred program that the participants had taken part in had a problem-oriented approach. It is plausible that for some participants a rehabilitation program with more focus on remaining competences might have been more beneficial. Furthermore, the program entailed that the OT and the participant together set rehabilitation goals. Despite this common procedure, all participants did not experience involvement in setting goals, although the procedure was ascertained in a review of the OTs’ medical records (Flink et al., 2016). Similar findings where all clients did not experience involvement in goal-setting have been reported in the context of client-centred practice (Walder & Molineux, 2020). Hence, the mechanisms for clients to experience partnership in goal setting and agency in rehabilitation need further exploration.

A limitation of the CADL program might be that it only involved one of the health professions commonly engaged in the rehabilitation team after stroke. We propose that applying a common client-centred approach in the rehabilitation team would be preferable for the experience of the client and plausibly leading to better outcomes. Hence, future studies of client-centred rehabilitation in which the whole team is involved are warranted.

This study is researching four stroke survivors’ experiences over one year after having participated in a client-centred occupational therapy program. One of the study’s strengths is that data were collected longitudinally, i.e., at three to four different points in time rendering 13 interviews with rich data allowing the authors to explore the emerging categories over time, as opposed to experiences over time collected at one point in time. In the latter case, recall bias of earlier experiences and changes in perspectives may be distorted or remain undetected.

The trustworthiness of the findings (Polit & B, 2017) builds on the authors’ systematic reflections, on the main categories and the sub-categories. Since the data were already collected prior to analysis, a limitation is that theoretical sampling i.e., the cyclical process of collecting and coding data after the first interview could not be performed limiting the possibility to gain a deeper knowledge in the emerging categories (Charmaz, 2006). That the same questions were asked at each point in time of data collection could on the other hand also be considered an advantage when comparing the responses of participants across time (Kneck & Audulv, 2019). Furthermore, the interviews were analysed separately by point in time at which they were collected, and the authors were open to new emerging categories at the separate points in time. Discussions between all authors supported openness to unexpected findings while keeping the original research questions in focus (Tuthill et al., 2020). Confirmability (Polit & B, 2017) was strengthened by the fact that the first author had not previously been involved in the client-centred ADL-program and was neutral in the analysis and interpretation. Thus, the categories were based on the participants’ narratives and not on the preunderstanding of the authors (Tuthill et al., 2020). During the analysis, categories and memos were iteratively discussed between the first and the last authors to improve resonance i.e., investigator triangulation (Carter et al., 2014). Furthermore, when focusing on changes across time (Tuthill et al., 2020) the authors systematically looked for similarities and differences in the data over the year by applying initial focused and axial coding to describe the processes throughout the year (. Transferability is strengthened by the fact that all participants were recruited in the same client-centred context, the CADL program, which has previously been described in detail (Bertilsson et al., 2014; Guidetti et al., 2020) and in the same hospital. However, this may also be a limitation since client-centred content and contexts will no doubt vary. Furthermore, the CADL program was completed by the time of the second interview. On the other hand, the aim of the CADL was to enable agency in daily activities and participation in everyday life after stroke, and with outcomes sustainable beyond the hospital stay.

It could be considered a limitation that all participants were men with left hemisphere lesions, and caution should be exerted when transferring our findings to other contexts, right hemisphere lesions and women, since women are reported to have poorer functional recovery, more depression and lower quality of life after stroke than men (Rexrode et al., 2022). On the other hand, it might also be considered a strength as the findings might be transferred to men, as was also the case in another scientific inquiry after stroke of men only (Van de Velde et al., 2016), and to men with left hemisphere lesions. Further studies of experiences of clients in client-centred rehabilitation contexts are warranted where women are included, as well as right hemispheres lesions, varying rehabilitation environments, e.g., hospitals and home environments.

### Ethical considerations

All participants in this study had recently had a stroke, a life-threatening event; hence, they were all in a vulnerable situation. To be interviewed and share reflections after such an event may cause sadness and suffering. The interviewer, an experienced clinician, could refer the clients for professional support when needed. Otherwise, based on our previous extensive experience in interviewing people with acquired brain injury, stroke survivors often express gratitude to be heard and that someone is interested in their lives and experiences. They furthermore often express that sharing is giving some meaning to their negative experiences, particularly if it generates new knowledge that in the future may help others in similar situations. Another ethical concern is that longitudinal studies with recurrent interviews by the same data collector are likely to create a relationship between the interviewer and the informant. As the relationship will be discontinued once the data collection is completed careful monitoring of engagement and support must be exerted throughout the study not to create feelings of abandonment. As in most research, it is vital to make sure that the identity of study participants remain unknown, yet it is also vital to supply information to the reader of the study participants’ characteristics. In this qualitative study, where the number of participants was small, the information of the study participants that could be supplied was limited. On the other hand, the information supplied is adequate, as the aim of the study was not to unveil explanations or associations of the participants’ experiences to their individual characteristics. Such knowledge is indeed of vital importance too but would require a different study design and sample of participants.

## Conclusion

While in hospital stroke survivors can have unrealistic expectations of what life would be when coming home. When at home they can realise their actual capacity in performing activities. To find a “new” self after a stroke can involve time-consuming and taxing processes of reflections of self-understanding related to having had a stroke, changes in private life and work. Engagement in self-selected meaningful and valued activities can support stroke survivors’ reconstruction of the “new” self but not all stroke survivors may succeed in finding their “new” self during the first year after stroke.

## Supplementary Material

Short biographical notes.docx
