# Supplementary material for: In search of self after stroke: a longitudinal qualitative study in the context of client-centred rehabilitation
Source: Int J Qual Stud Health Well-being. 2023 Nov 27;18(1):2282513. doi: 10.1080/17482631.2023.2282513 (PMC11000675; doi:10.1080/17482631.2023.2282513)
Supplement: Short biographical notes.docx [file ZQHW_A_2282513_SM5851.docx]

**Short biographical notes**

Anette Erikson, PhD is an occupational therapist. Anette Erikson’s research is targeting the lived experiences of people with stroke and their families.

[Maria Ranner,](https://www.ltu.se/staff/m/marran-1.122893?l=en) PhD, is a senior lecturer in occupational therapy at Luleå University of Technology. The focus of the Dr Ranner’s research is on re-ablement and client-centred rehabilitation.

[Susanne Guidetti,](https://staff.ki.se/people/susanne-guidetti) PhD, is professor in Occupational Therapy at Karolinska Institutet. Professor Guidetti’s research is focused on developing and evaluating complex interventions related to health promotion and prevention, interventions, education in occupational therapy, as well as with a broader interdisciplinary strategy. The research is conducted both in high- and low-income countries.

[Lena von Koch,](https://staff.ki.se/people/lena-von-koch) PhD is professor in Health Services Research at Karolinska Institutet. Professor von Koch’s has long experience of research in the development and evaluation of health services for people with neurological disabilities and their families. Professor von Koch’s research involve the stakeholders in the co-creation of improved health services e.g. care transitions after stroke. The research is conducted both in high- and low -income countries.
